# PTEN breast cancer susceptibility: a matter of dose

**DOI:** 10.3332/ecancer.2010.192

**Published:** 2010-10-07

**Authors:** A Alimonti

**Affiliations:** Laboratory of Experimental Oncology, Oncology Institute of Southern Switzerland, via Vela 6, 6500 Bellinzona, Switzerland; Fatebenefratelli Research Center, San Pietro Fatebenefratelli, via Cassia 600, 00189, Rome, Italy

## Abstract

The phosphatase and tensin homolog located on chromosome ten, PTEN, is one of the most commonly mutated tumor suppressor genes (TSGs) in human cancer [1–3]. PTEN catalyzes the conversion of the membrane lipid second messenger PIP3 to PIP2 and is therefore a key mediator of the AKT/PKB pathway [4,5]. Although inherited PTEN mutations predispose to the development of Cowden syndrome, which is also a breast cancer susceptibility syndrome, the role of PTEN in breast tumorigenesis has been considered minor when compared to that of other TSGs such as BRCA1 or p53 [6]. There is no current evidence that mutations in PTEN account for a substantial proportion of familial breast cancer in the absence of Cowden syndrome [6]. Moreover, PTEN mutations or deletions are not common in sporadic breast tumors, especially when compared with other tumor types (<5%) such as prostate cancer [7, 8].

Despite this evidence, recent studies have demonstrated that PTEN protein down-regulation is frequently observed (more than 50%) in sporadic breast tumors, highlighting the relevance of the dose of this TSG for the pathogenesis of breast cancer [7–9]. Our paper, in the last month’s issue of *Nature Genetics* provides additional evidence of the role of PTEN dose in breast cancer susceptibility, braking current dogmas regarding the development of cancer and opening to novel clinical and therapeutic implications [10].

## Challenging the Two Hit hypothesis of tumorigenesis

For students that, like myself, attended medical school in the mid 1990s, it was often unclear, with few exceptions, how novel discoveries in cancer genetics could impact on the clinic. At that time, the mechanism driving tumor initiation and cancer progression was reasonably clear. One of the first lessons in the class of cancer genetics included the explanation and discussion of the well known Knudson’s two-hit model of tumorigenesis [11]. Knudson envisioned tumor suppression as a recessive trait, whereby an inactivating mutation present at birth is accompanied by a second somatic loss in the tumor. His findings in patients affected with retinoblastoma were entirely in agreement with his proposed model of tumorigenesis [11]. As a result of this visionary working model, a number of TSGs that undergo bi-allelic disruption or truncating point mutations were identified. Only later, in the era of mouse genetics was it demonstrated that for some TSGs such as p53 even inactivation of a single allele of the TSG would have been sufficient to initiate and promote tumorigenesis [12]. The genes that behave as p53 were named “haploinsufficient” tumor suppressor genes.

**Figure 1:**
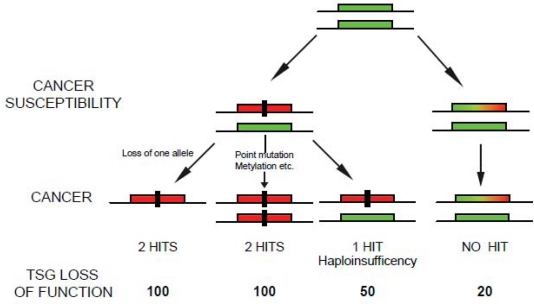
Implications for tumorigenesis upon subtle reduction of TSG levels. The ‘no hits’ model of cancer susceptibility. The rectangular green box represents a functional allele of a given TSG. The rectangular red box represents a nonfunctional allele inactivated by, for example, mutation or deletion. The rectangular green, yellow and red box represents an allele of a TSG whose expression is reduced below the normal levels. The black rectangle represents a genetic hit. Note that the model represented does not exclude the presence of additional hits on other loci.

**Figure 2:**
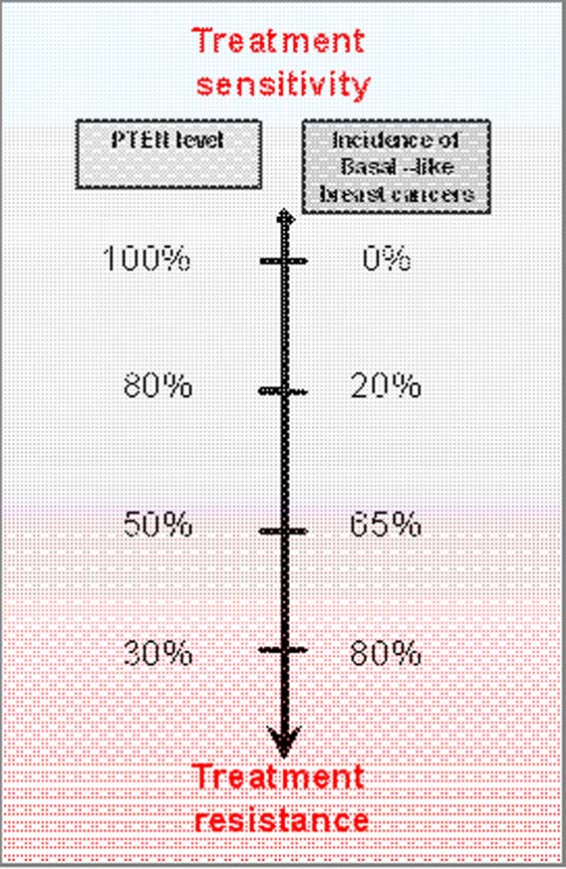
Correlation between PTEN levels, incidence of basal-like breast tumors and treatment resistance. Note that the progressive reduction of PTEN levels is inversely correlated with the incidence of basal like breast tumors.

In our recent paper in *Nature Genetics*, we push further the concept of haploinsufficiency demonstrating that even subtle changes in the level of PTEN can significantly increase the chance of developing breast cancer [10]. Mice with *Pten* expression below 20% the normal expression levels develop breast tumors in absence of additional mutations or loss of heterozigosity (LOH) in the *Pten* alleles [10]. Importantly, this subtle reduction in Pten levels initiate breast cancer susceptibility without consequence for other tissues including the prostate epithelium [10]. On this line, we observed that the progressive reduction of Pten levels in the mouse population was associated with the development of highly aggressive breast tumors and with the appearance of additional tumor phenotypes [10]. Mice born with 50% of Pten levels (*Pten*^+/−^) develop basal like breast cancers at higher frequency than mice born with 80% of Pten (*Pten*^hy/+^) (Fig.2). In addition, Pten^+/−^ mice developed prostatic intraepithelial neoplasia and uterine cancer, which we did not observe in the *Pten*^hy/+^ population [10]. This suggests that some tissues can be more susceptible than others to minimal variations of Pten levels and worn us on the relevance of gene doses rather than gene mutations for cancer initiation and progression. Our study adds another dimension to the “Knudsonian model of tumorigenesis” demonstrating that cancer susceptibility can be driven by a progressive —but slight—continuum reduction in tumor suppressor levels. This implies that any factors —chemicals, diet, and other carcinogens— which affect the level of a TSG without affecting its genetic structure are potential “pro-oncogenic” factors. In our paper, we propose a continuum working model for cancer initiation in which subtle variations in the expression of TSGs have a profound impact on cancer susceptibility [11] (Fig. 1). This model differs from previous models of tumorigenesis which imply the progressive accumulation of mutations in a “saltatory” manner.

## Clinical and therapeutic implications of PTEN dose

PTEN deficiency not only drives breast cancer tumorigenesis but also confers resistance to targeted therapy with trastuzumab, a humanized monoclonal antibody against ErbB2 (also referred to as HER2/neu). Trastuzumab is the most effective treatment offered to metastatic breast cancer patients with HER2 over expression [9]. Binding to the ErbB2 receptor, Trastuzumab stabilizes and activates the tumor suppressor PTEN and consequently down-regulates the PI3K–Akt signaling pathway. When PTEN levels are down-regulated the effects of trastuzumab are impaired and the outcome and prognosis of patients decrease [9]. Our previous and recent findings further demonstrate that PTEN protein down-regulation leads to the development of basal-like breast tumors [8]. This tumor phenotype is associated with aggressive behavior, poor prognosis, and tumors typically lacking in the expression of hormone receptors or HER-2 (“triple-negative” phenotype) [8]. Thus, patients with basal-like cancers are unlikely to benefit from trastuzumab treatment, as well as from hormonal and conventional chemotherapy.

Despite this evidence, the assessment of PTEN status in breast cancer patients undergoing treatment with targeted or conventional therapy is still not commonly recommended by clinicians. Indeed, the percentage of patients with *PTEN* mutations is low and not sufficient to justify screening for *PTEN* mutations/deletions. In addition, the determination of PTEN protein levels depends on the reliability of PTEN immunohistochemistry and the ability of the pathologists.

Our novel findings suggest that subtle variations in PTEN expression may also influence the outcome of patient treatments and point to the assessment of PTEN expression rather that PTEN mutations or protein determination in patients undergoing targeted or conventional treatments [10] (Fig.2).

Interestingly, in our paper we show that in all the patients affected with basal like- breast tumors, *PTEN* expression is down-regulated when compared to controls [10]. Moreover, we demonstrate that the immunohistochemistry for PTEN is not a reliable means to identify subtle variations of PTEN in tumor. Samples where PTEN expression was subtly down-regulated scored positive on the immunohistochemistry and it was not possible to discriminate between controls and tumor samples with moderate/slight decrease in PTEN [10]. Therefore, it is possible to hypothesize that, previous studies where PTEN levels were evaluated by immunohistochemistry have underestimated the role of this TSG in predicting the outcome of cancer therapy. Hence, highly quantitative combinatorial methods for integrating genomic and proteomic information are required to precisely determine the impact of such subtle down-regulations in PTEN expression on both targeted and individualized approaches for cancer therapy. We believe that a positive future development of our findings will be the identification of genetic factors or novel PTEN interacting proteins, which affecting directly or indirectly the level of PTEN expression may initiate breast cancers predisposition or affect the outcome of cancer therapy. An example of this could be the identification of single nucleotide polymorphisms in the promoter of PTEN or the discovery of negative regulators of the transcription or degradation of this TSG. This may lead to the development of novel genetic tests for the screening of subjects with high breast cancer risk or that may be used to predict the outcome of cancer therapy.
